# Housed feeding improves rumen health by influencing the composition of the microbiota in Honghe cattle

**DOI:** 10.3389/fvets.2025.1556934

**Published:** 2025-03-10

**Authors:** Runqi Fu, Lin Han, Chunjia Jin, Ye Yu, Binlong Fu, Qian Li, Jing Leng

**Affiliations:** ^1^Yunnan Provincial Key Laboratory of Animal Nutrition and Feed, Yunnan Agricultural University, Kunming, China; ^2^Faculty of Animal Science and Technology, Yunnan Agricultural University, Kunming, China

**Keywords:** cattle, antioxidant, immunity, rumen barrier function, rumen microbiota

## Abstract

Rumen is one of the most vital organs for the digestion of ruminants and is influenced by factors including feeding patterns and nutrition. How rumen microbiota and barrier function change are affected feeding patterns requires attention, particularly for beef cattle. In the present study, the Honghe cattle under grazing (CON group, *n* = 10) and housed feeding (HES group, *n* = 10) conditions were selected as a model of different rumen microbiota and observed for 180 days. The indicators of immunity and antioxidants in serum and rumen epithelium of cattle were measured; and the rumen microbiota were evaluated by using 16S rRNA and ITS sequencing techniques. In the present study, the concentrations of total protein, albumin and glucose in serum of Honghe cattle were significantly increased by the HES group when compared with CON group (*p* < 0.05). The HES group reduced the levels of complement 3, complement 4, interleukin-4, interleukin-10, interleukin-1β and tumor necrosis factor *α*, but increased the levels of total antioxidant capacity (T-AOC) and superoxide dismutase (SOD) (*p* < 0.05). We found that the HES group enhanced the levels of T-AOC and SOD in rumen epithelium (*p* < 0.05). Furthermore, there was a significant up-regulation of the relative mRNA expressions of *ZO-1*, *OCC*, *SOD1*, *SOD2*, *Nrf2*, *NQO-1* and *HO-1* observed in the HES group (*p* < 0.05). For rumen microbiota, the HES group significantly decreased alpha diversity. The core rumen bacterial communities were Bacteroidata, Firmicutes and Proteobacteria. The relative abundances of *Prevotella* and *Ruminococcus* were increased by the HES group, but *norank_f_Bacteroidales_UCG-001*, *Rikenellaceae_RC9_gut_group* and *Prevotellaceae_UCG-003* were decreased (*p* < 0.05). Moreover, The HES group enhanced the relative abundance of *Pichia*, *Cyllamyces*, *Sterigmatomyces* and *Wallemia* (*p* < 0.05), but decreased *Aspergillus* and *Candida* (*p* < 0.05). There was a positive correlation between microorganisms such as *Prevotella*, *Ruminococcus* and *Pichia* and rumen epithelial barrier and antioxidant-related genes (*p* < 0.05). Overall, housed feeding contributed to the improvement of antioxidant capacity and rumen health in Honghe cattle, which may be related to the modulation of rumen microbiota including bacteria and fungi.

## 1 Introduction

In wild and free-range cattle, various grazing factors can adversely affect health, contingent upon conditions such as pasture nutrition, feeding practices and weather (1). For instance, overgrazing often results in protein metabolism imbalances and energy deficiencies, raising health concerns (1–3). Additionally, direct exposure to heat and cold stress elevates stress hormone levels, thereby suppressing immune function (4). These findings indicated that feeding practices were intricately linked to multiple facets of animal health, including behavior, physiology, nutrition, and immune status (5). Recently, the beef cattle industry has increasingly shifted toward intensification and scaling up to meet human demands. Cattle that previously inhabited variable environments, such as high altitudes, mountainous terrains, or areas with fluctuating temperatures, are now transitioning to more stable and homogenized conditions (6, 7). Consequently, feeding practices have altered physiological activities, including growth and development, digestion, and metabolism, with significant impacts on immunity and antioxidant capacity in cattle reared in uniform environments. The management of animals generally transitions from a traditional grazing system to a semi-grazing and semi-captive system, and then further to a complete housed feeding (8). The effects on the physiological status of animals are not yet clear when the transition is made directly from grazing to housed feeding. Previous studies on the effects of feeding practices on animal health have assessed only a few parameters, such as peripheral blood neutrophil and monocyte counts (9), cortisol (5), and total antioxidant capacity (1, 8). However, an integrated evaluation of the effects of feeding practices on animal health requires a comprehensive analysis of multiple parameters in combination with tissue expression levels.

It is worth noting that the rumen health of cattle will receive more attention in the context of changes in management practices and nutrient availability, among other things. The rumen system involves physical and chemical barriers in the rumen epithelium, immune and antioxidant defense mechanisms, etc., as well as a complex rumen microbial community that can convert indigestible plants into nutrients and energy (10, 11). Collectively, these determine the health of the rumen in the face of changes in the internal or external environment. Previous studies have demonstrated that rumen community composition was susceptible to dietary or feeding practices (12). For example, the rumen diversity was higher in grazing yaks than in housed yaks (13). By contrast, the housed fattening system provided more nutrients that favored yak growth performance and rumen development (6). Moreover, the rumen epithelial barrier effectively defends against substances including antigens and free radicals, and controls nutrient absorption processes (10). However, to date, there is a limited understanding of changes in barrier function and the microbial composition of the rumen health and feeding system changes in cattle. A comprehensive analysis of rumen health could provide important insights into livestock production. Understanding the impact of feeding patterns on rumen health has important implications for healthy and efficient ruminant production. Honghe cattle, also known as Honghe Yellow cattle, is a representative local cattle breed in Yunnan, China (14). It is currently raised in grazing, semi-grazing or housed-feeding mode in the highland areas, and has been developed toward large-scale farming. However, the mechanisms by which feeding patterns influence the rumen health of Honghe cattle remain largely unknown.

Therefore, considering the above, we hypothesized that feeding practices would affect immune and antioxidant status and rumen health in cattle. This study assessed the potential impact of changes in feeding practices by comparing the immune response, oxidative status, rumen barrier function and microbiological composition of grazing and housed Honghe cattle.

## 2 Materials and methods

### 2.1 Animals and sample collection

A total of twenty healthy male Honghe cattle (two-year-old) with an average weight of 254.53 ± 19.69 kg were selected for this experiment, and were randomly allotted to two groups (10 replicates per group and one cattle per replicate). The Honghe cattle in the control (CON) group grazed only on natural pastures without any supplements, while the housed (HES) group was fed with total mixed ration (TMR). The basic components of the TMR were presented in Supplementary Table S1. The HES group of cattle were fed in a semi-open barn and individually housed in 5 × 4.5 m indoor pen. The CON group of cattle were moved in the adjacent pasture. The cattle in the CON group were grazed daily from 08:00–19:00, whereas those in the HES group were fed TMR twice a day (08:00 and 17:00), and all cattle had free access to feed and water. The experiment lasted for 180 days. At the end of the experiment, all the cattle were fasted for 24 h. The special immobilization frame was used to immobilize the cattle and then blood was collected from the jugular vein. Blood samples were centrifuged at 3500 r/min for 10 min at 4°C to separate the serum. Then, serum samples were stored at −80°C to prepare for analysis of serum biochemical, antioxidant and immunological indexes. Next, all cattle were humanely harvested at a commercial slaughterhouse. Ruminal epithelial samples were taken from the same location in the lower part of the rumen, washed with sterile PBS and excess water was removed with filter paper, then placed in 2 mL freezing tubes and immediately stored in liquid nitrogen. Rumen fluid samples were filtered through four layers of gauze and placed in 5 mL freezing tubes and immediately stored in liquid nitrogen. The rumen epithelial samples were used for mRNA sequence analysis, while the rumen fluid samples were used to analyze microbial composition and structure.

### 2.2 Determination of biochemical, antioxidant and immunological indexes in serum and rumen epithelium

Blood samples were analyzed for serum biochemical, antioxidant and immunological indexes. According to the methods of Fu et al. (15), the concentrations of total triglycerides (TG; glycerol phosphate oxidase-p-aminophenazone method), total cholesterol (TC; glycerol phosphate oxidase-p-aminophenazone method), total protein (TP; Bradford method), albumin (ALB; bromocresol green method), urea nitrogen (BUN; urease method), glucose (GLU; glucose oxidase method), high-density lipoprotein cholesterol (HDL-C; colorimetry), low-density lipoprotein cholesterol (LDL-C; colorimetry) in serum were determined using the corresponding commercial kits (Nanjing Jiancheng Bioengineering Institute, Nanjing, China). The antioxidant indicators in serum including total antioxidant capacity (T-AOC), superoxide dismutase (SOD), malondialdehyde (MDA) and glutathione peroxidase (GSH-Px) were assayed using commercial kits (Nanjing Jiancheng Bioengineering Institute, Nanjing, China) corresponding to methods ABTS, WST-1, TBA and colorimetric, respectively. The following indicators were measured using commercially available enzyme-linked immunosorbent assay (ELISA) kits (Jiangsu Meimian Industrial Co., Ltd., Jiangsu, China) according to the instructions: immunoglobulin A (IgA), immunoglobulin G (IgG), complement 3 (C3), complement 4 (C4), interleukin-4 (IL-4), interleukin-10 (IL-10), interleukin-1β (IL-1β), tumor necrosis factor *α* (TNF-α). In addition, the 200 mg sample of rumen epithelium was homogenized to obtain a suspension, which was further centrifuged (3,500 r/min, 10 min, 4°C) to obtain the supernatant for analysis the levels of IL-4, IL-10, IL-1β, TNF-*α*, T-AOC and MDA as well as the activity of SOD and GSH-Px.

### 2.3 RNA extraction and mRNA abundance analysis

Total RNA was extracted from the samples of rumen epithelium of each Honghe cattle using TRIzol reagent (TaKaRa Biotechnology, Otsu, Japan). The concentration of total RNA was determined by the Nanodrop 2000 spectrophotometer (Thermo Fisher Scientific, Waltham, MA). The quality was identified by the agarose gel electrophoresis on a horizontal electrophoresis apparatus (Bio-Rad Laboratories, Richmond, CA). Then, complementary DNA (cDNA) was reverse-transcribed by a PrimeScript™ FAST RT reagent kit (TaKaRa) according to the manufacturer’s introduction. The real-time fluorescence quantitative PCR was performed using the TB Green Premix Ex Taq reagents (TaKaRa) via the CFX-96 RT-qPCR Detection System (Bio-Rad). To target the genes related to the rumen barrier, development and absorption, all primers were synthesized by Invitrogen (Shanghai, China) and presented in Supplementary Table S2. The *ACTB* was used as a housekeeping gene since it was not affected by experimental factors. The relative expression of all target genes was calculated with reference to Arce et al. (16) using 2^-△△^Ct method.

### 2.4 DNA extraction and microbial structure

The composition and structure of rumen microbiota were analyzed from two perspectives, bacteria and fungi, respectively. Specifically, the total genomic DNA of rumen fluid samples was extracted by the E.Z.N.AR^®^ kit (OmegaBio-tek, Norcross, GA, UnitedStates) following the manufacturer’s instructions. The quantity and quality of total DNA were examined using a Nanodrop 2000 spectrophotometer and 1% agarose gel electrophoresis, respectively. After the V3-V4 region of 16S rRNA was amplified (338F, ACTCCTACGGGGAGGCAGCAG and 806R, GGACTACHVGGGTWTCTAAT) and the agarose gel was opened, the 16S rRNA was purified by QIAquick Gel Extraction Kit (Qiagen, Hilden, Germany) on the bands were purified. Similarly, the fungus was sequenced using an internal transcribed spacer region (ITS) with the amplification region ITS1F-ITS2R (upstream, CTTGGTCATTTAGAGGAAGTAA, downstream, GCTGCGTTCTTCATCGATGC). It was then sequenced by Shanghai Majorbio Bio-Pharm Technology Co. Ltd. (Shanghai, China) using the Illumina MiSeqPE250 platform. The reaction conditions and procedures for PCR amplification of the 16S rRNA and ITS1 genes followed the study of Liu et al. (17). The quality filtering, clustering and analysis of sequencing data were performed as described by the method of Wu et al. (18).

### 2.5 Statistical analysis

Data from both serum and rumen epithelium were analyzed using SAS statistical software (version 9.4; S.A.S, Institute Inc., Cary, NC, USA) and normality distribution was tested using the Shapiro–Wilk test. Then, the above data were analyzed using two-tailed Student’s t-test and each cattle served as a statistical unit. Microbial relative abundance data from 16S rRNA and ITS sequencing were analyzed using Kruskal-Wallis one-way ANOVA. Spearman rank correlation coefficients were employed to assess the correlation between rumen microbiota and the barrier, antioxidant and immune-related genes. Statistical significance of all data was considered at *p* < 0.05, with 0.05 ≤ *p* < 0.10 representing a trend.

## 3 Results

### 3.1 Serum biochemical parameters

**Figure 1:**
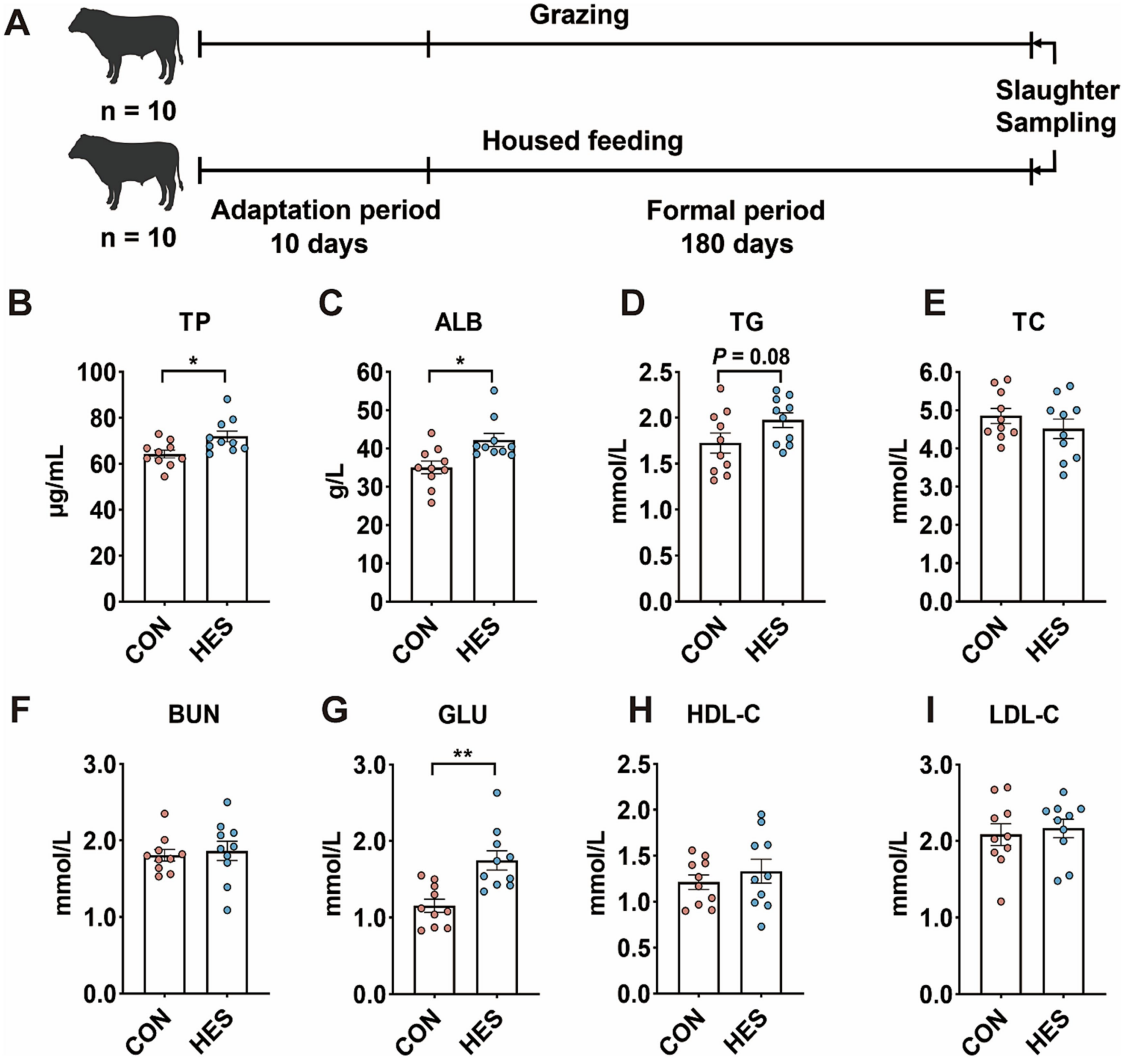
Effect of housed feeding pattern on serum biochemical parameters of Honghe cattle. **(A)** Schematic diagram of the feeding experiment and sample collection. **(B)** Total protein (TP). **(C)** Albumin (ALB). **(D)** Total triglycerides (TG). **(E)** Total cholesterol (TC). **(F)** Urea nitrogen (BUN). **(G)** Glucose (GLU). **(H)** High-density lipoprotein cholesterol (HDL-C). **(I)** Low-density lipoprotein cholesterol (LDL-C). Grazing group, i.e., control group (CON). Housed feeding group (HES).

As shown in Figure 1, the HES group had higher concentrations of TP (Figure 1B) (*p* < 0.05), ALB (Figure 1C) (*p* < 0.05) and GLU (Figure 1G) (*p* < 0.01) compared to the CON group. HES group tended to enhance TG level (Figure 1D) (*p* = 0.08). There were no significant differences in serum levels of TC (Figure 1E), BUN (Figure 1F), HDL-C (Figure 1H) and LDL-C (Figure 1I) between HES and CON groups (*p* > 0.05).

### 3.2 Immunological and antioxidant parameters in serum

**Figure 2:**
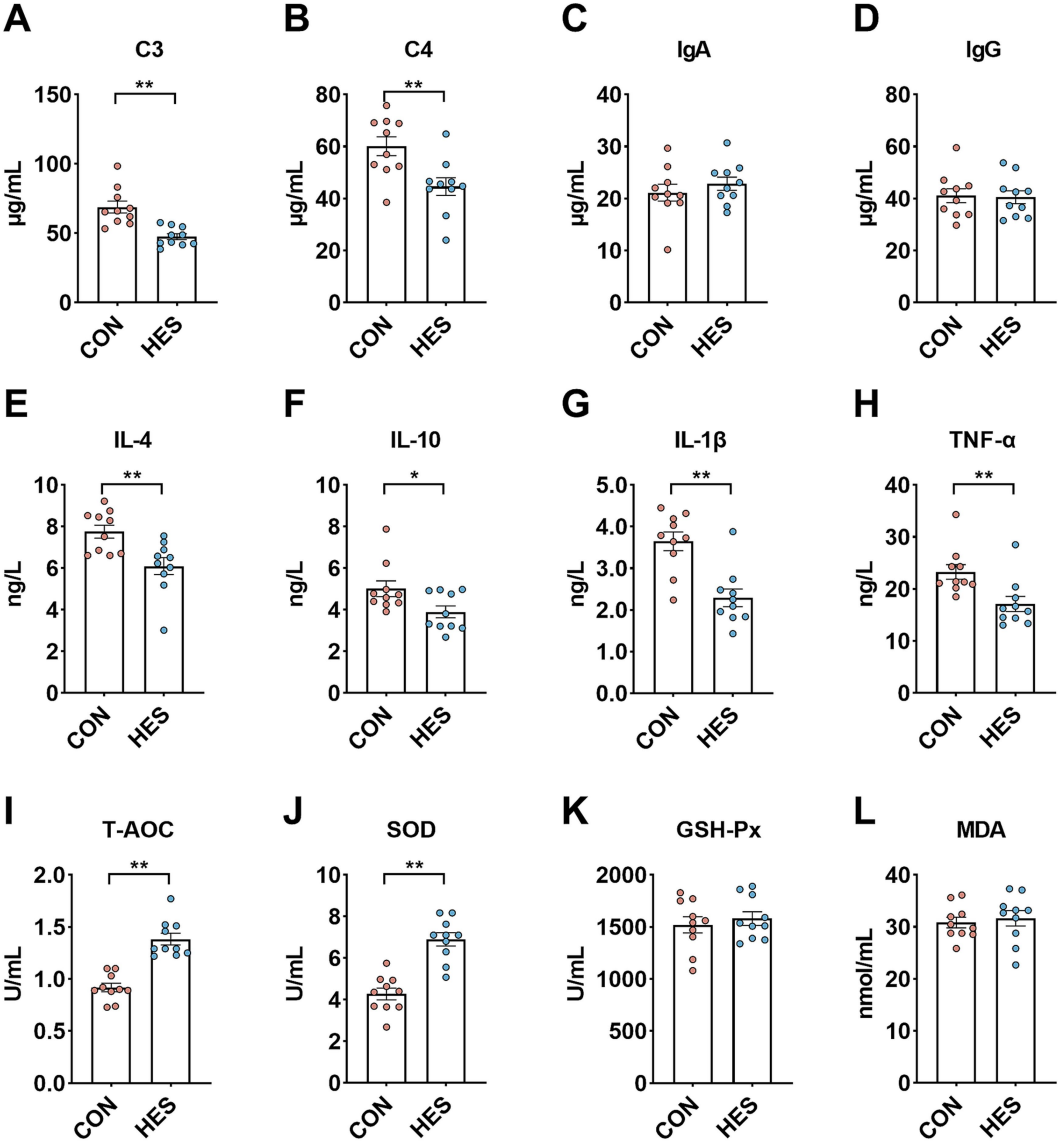
Effect of housed feeding pattern on immune and antioxidant indices in serum of Honghe cattle. **(A)** Complement C3 (C3). **(B)** Complement C4. **(C)** Immunoglobulin A (IgA). **(D)** Immunoglobulin M (IgM). **(E)** Interleukin-4 (IL-4). **(F)** Interleukin-10 (IL-10). **(G)** Interleukin-1β (IL-1β). **(H)** Tumor necrosis factor *α* (TNF-α). **(I)** Total antioxidant capacity (T-AOC). **(J)** Superoxide dismutase (SOD). **(K)** Glutathione peroxidase (GSH-Px). **(L)** Malondialdehyde (MDA). Grazing group, i.e., control group (CON). Housed feeding group (HES).

To evaluated the effects of housed feeding on the systemic immune and antioxidant capacity of Honghe cattle (Figure 2), we observed that C3 (Figure 2A) and C4 (Figure 2B) were significantly lower in serum of group HES than in CON (*p* < 0.05). For cytokines in serum, the HES group had lower concentrations of IL-4 (Figure 2E), IL-10 (Figure 2F), IL-1β (Figure 2G) and TNF-*α* (Figure 2H) than the CON group (*p* < 0.05). Furthermore, the levels of T-AOC (Figure 2I) and the activity of SOD (Figure 2J) in serum were significantly higher when the HES group compared with the CON group (*p* < 0.05). There was no difference in the concentration of MDA (Figure 2L) and the activity of GSH-Px (Figure 2K) between the CON and HES groups (*p* > 0.05).

### 3.3 Immunological and antioxidant capacity in rumen epithelium

**Figure 3:**
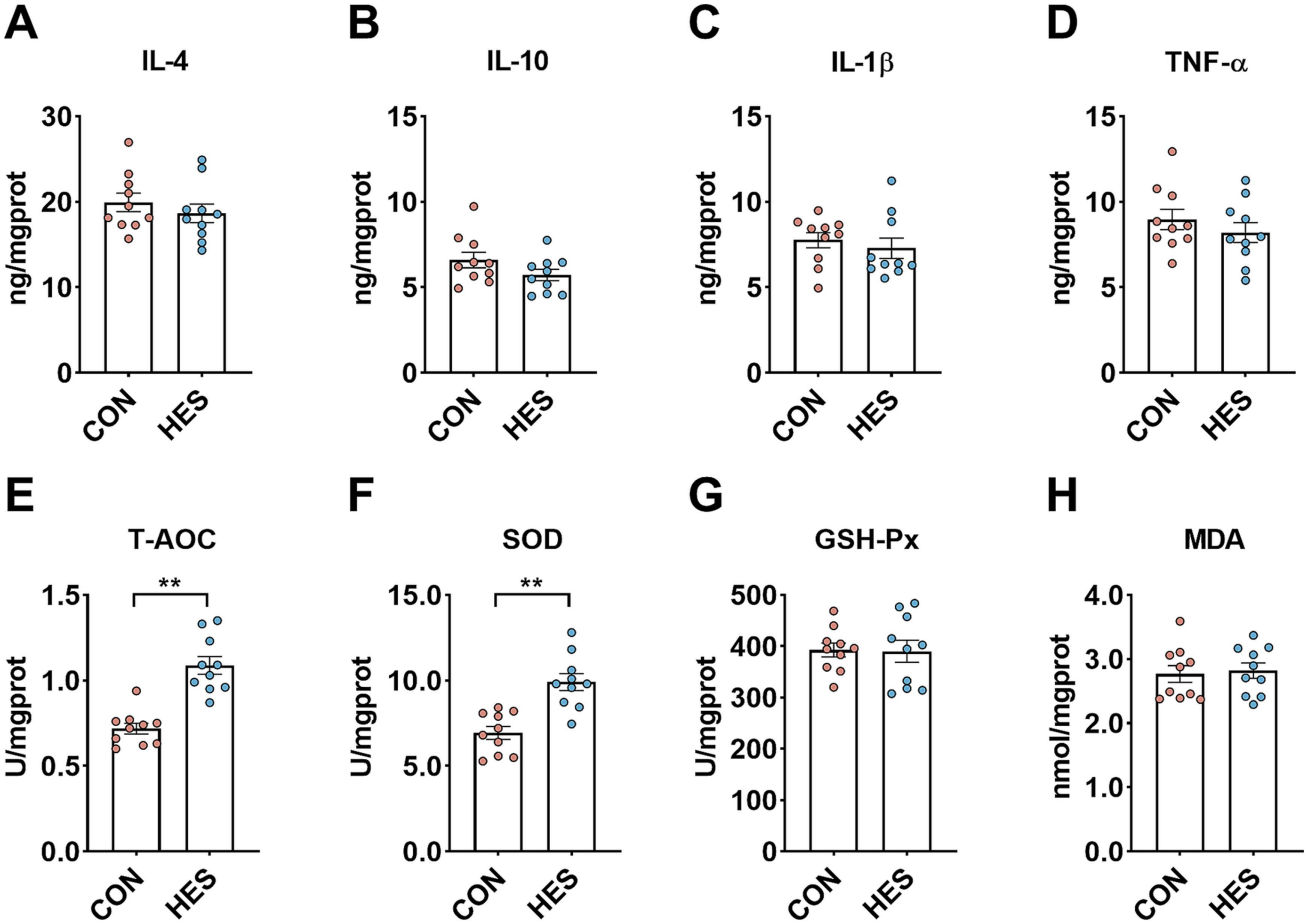
Effect of housed feeding pattern on immune and antioxidant indices in the rumen epithelium of Honghe cattle. **(A)** Interleukin-4 (IL-4). **(B)** Interleukin-10 (IL-10). **(C)** Interleukin-1β (IL-1β). **(D)** Tumor necrosis factor α (TNF-α). **(E)** Total antioxidant capacity (T-AOC). **(F)** Superoxide dismutase (SOD). **(G)** Glutathione peroxidase (GSH-Px). **(H)** Malondialdehyde (MDA). Grazing group, i.e., control group (CON). Housed feeding group (HES).

We further analyzed the immunological and antioxidant capacity of the rumen epithelium (Figure 3). When compared with the CON group, there was no significant influence in the concentrations of IL-4 (Figure 3A), IL-10 (Figure 3B) and IL-1β (Figure 3C) in the rumen epithelium of the HES group (*p* < 0.05). The HSE group increased the levels of T-AOC (Figure 3E) and SOD (Figure 3F) when compared with CON group (*p* < 0.05), while there were no influences on the levels of GSH-Px (Figure 3G) and MDA (Figure 3H) (*p* > 0.05).

### 3.4 Barrier function of in rumen epithelium

**Figure 4:**
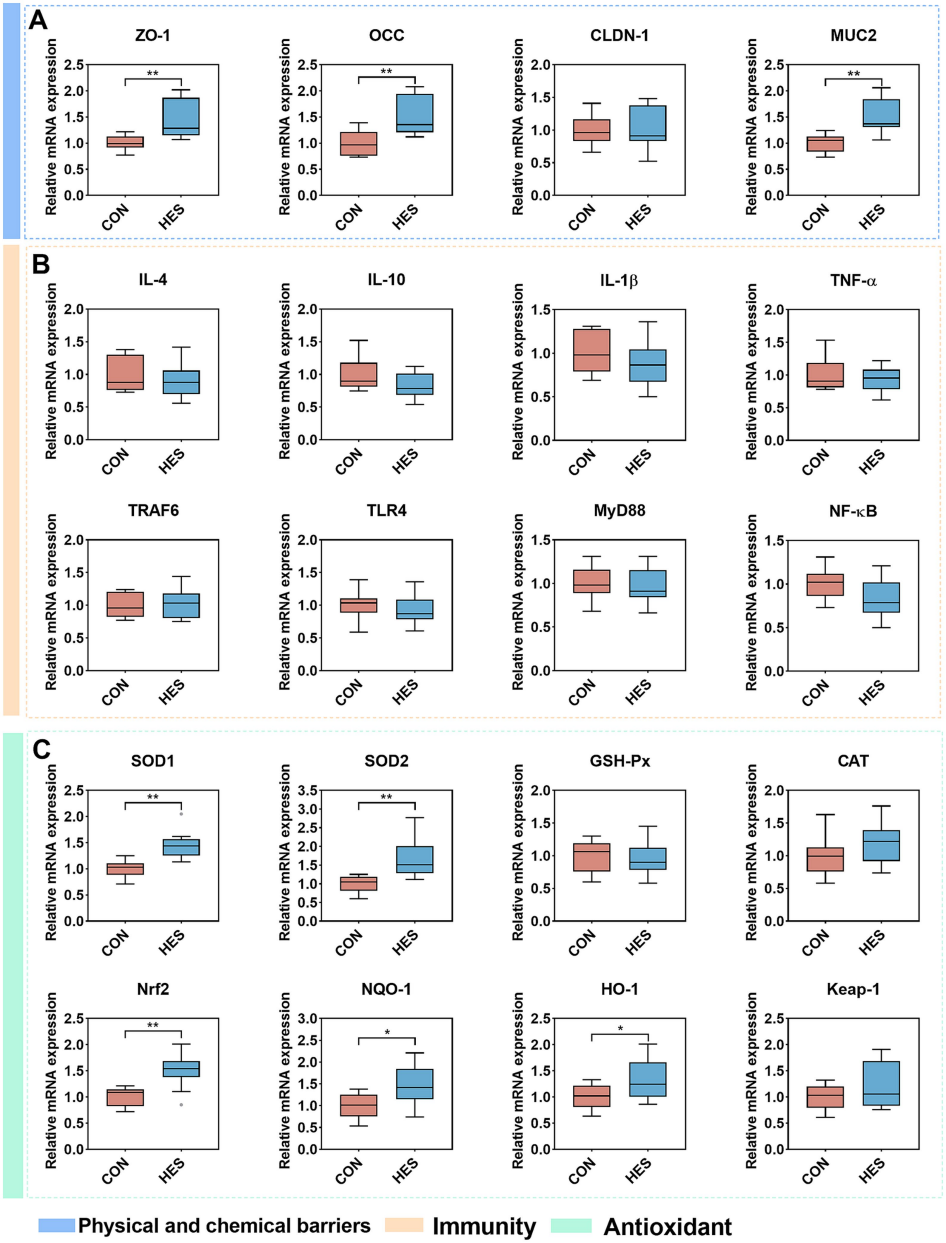
Effect of housed feeding pattern on the expression of rumen epithelial barrier-related genes in Honghe cattle. **(A)** Physical and chemical barrier-related genes. Zonula occludens-1 (ZO-1), occludin (OCC), claudin-1 (CLDN-1) and mucin2 (MUC2). **(B)** Immune-related genes. Interleukin-4 (IL-4), interleukin-10 (IL-10), interleukin-1β (IL-1β), tumor necrosis factor α (TNF-α), TNF receptor associated factor 6 (TRAF6), toll-like receptor 4 (TLR4), myeloid differentiation factor-88 (MyD88) and nuclear factor kappa-B (NF-κB). **(C)** Antioxidant-related genes. Superoxide dismutase 1 (SOD1), superoxide dismutase 2 (SOD2), glutathione peroxidase (GSH-Px), catalase (CAT), nuclear factor erythroid 2-related factor 2 (Nrf2), NAD(P)H:quinone oxidoreductase 1 (NQO-1), heme oxygenase-1 (HO-1) and kelch-like-ECH associated protein-1 (Keap-1). Grazing group, i.e., control group (CON). Housed feeding group (HES).

At present in Figure 4, the HES group significantly up-regulated the relative mRNA expressions of *ZO-1* and *OCC* in the rumen epithelium when compared with the CON group (Figure 4A) (*p* < 0.01). No significant differences were observed in the expressions of *IL-4*, *IL-10*, *IL1β* and *TNF-α* between groups HES and CON (Figure 4B) (*p* < 0.05). Additionally, there were no significant changes in the relative abundance of upstream signals *TRAF6*, *TLR4*, *MyD88* and *NF-κB* of cytokines formation (*p* > 0.05). Notably, the relative expression of antioxidant factors including *SOD1*, *SOD2*, *Nrf2*, *NQO-1* and *HO-1* was significantly up-regulated in the HES group (Figure 4C) (*p* < 0.05).

### 3.5 Bacterial composition in rumen

**Figure 5:**
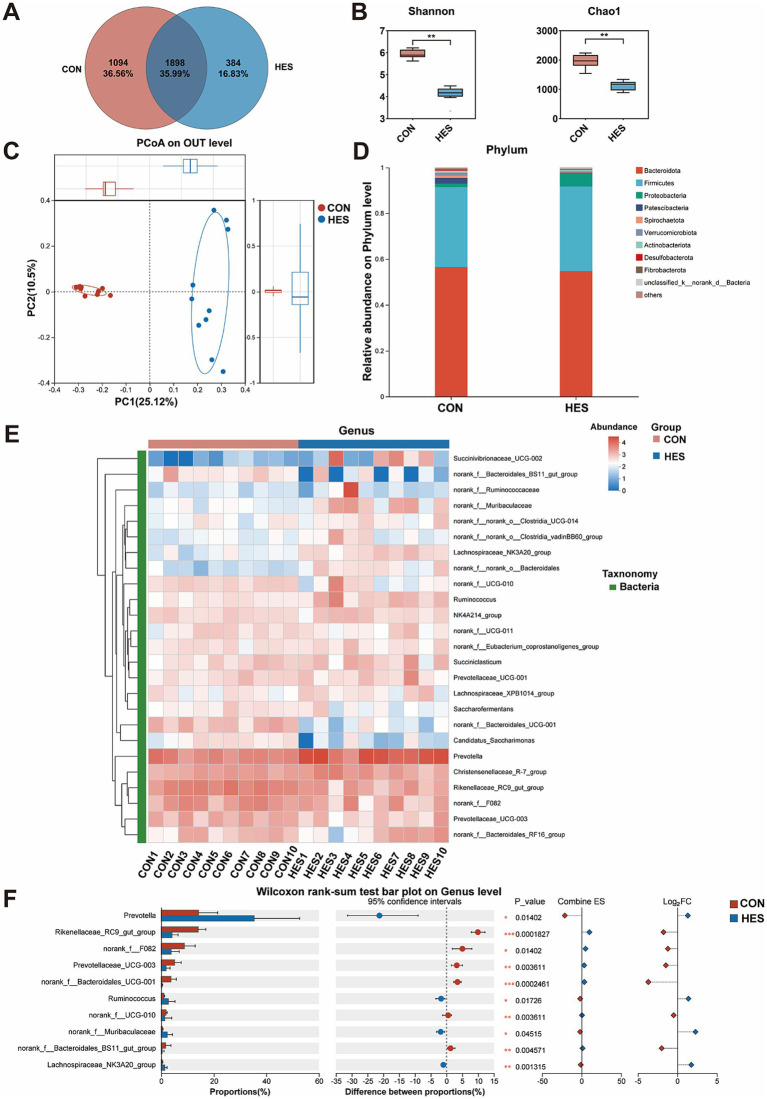
Effects of housed feeding patterns on the structure and composition of bacterial communities in the rumen of Honghe cattle. **(A)** OUT Venn diagram. **(B)** α-diversity. **(C)** PCoA. **(D)** Map of bacterial abundance at the phylum level. **(E)** Heatmap of Top 25 bacteria at genus level. **(F)** Differences in genus level of bacteria. Grazing group, i.e., control group (CON). Housed feeding group (HES).

To determine the effects of housed feeding on rumen health, we analyzed the composition of the rumen microbiota (Figure 5). According to 16S rRNA sequencing, a total of 3,376 OTUs were identified in the CON and HES groups, with 1,094 OTUs in the CON group and 384 OTUs in the HES group, and 1898 OTUs were common to both groups (Figure 5A). The α-diversity analysis showed differences in bacterial diversity between the two groups (Figure 5B), as evidenced by higher Shannon and Chao1 indexes in the HES group (*p* < 0.05). A significant difference in rumen bacterial counts between the HES and CON groups was observed according to PCoA (Figure 5C). We analyzed the classification of the 10 bacterial phyla identified. The rumen microorganisms of Honghe cattle in the CON and HES groups were dominated by three dominant phyla, namely Bacteroidata, Firmicutes and Proteobacteria, at the phylum level (Figure 5D). For the relative abundance of Firmicutes, Bacteroidetes and Proteobacteria, there were no significant differences between the CON and HES groups (*p* > 0.05). We selected the Top 25 bacteria in relative abundance at the genus level for analysis and observed several differential bacteria between the HES and CON groups (Figure 5E). Specifically, the HES group significantly enhanced the relative abundance of *Prevotella*, *Ruminococcus*, *norank_f_Muribaculaceae* and *Lachnospiraceae_NK3A20_group* (*p* < 0.05), but decreased the relative abundance of *norank_f_Bacteroidales_UCG-001*, *Rikenellaceae_RC9_gut_group*, *Prevotellaceae_UCG-003*, *norank_f_UCG-010*, *norank_f_Bacteroidales_BS11_gut_group*, *Lachnospiraceae_NK3A20_ group* and *norank_f_F082* (Figure 5F) (*p* < 0.05).

**Figure 6:**
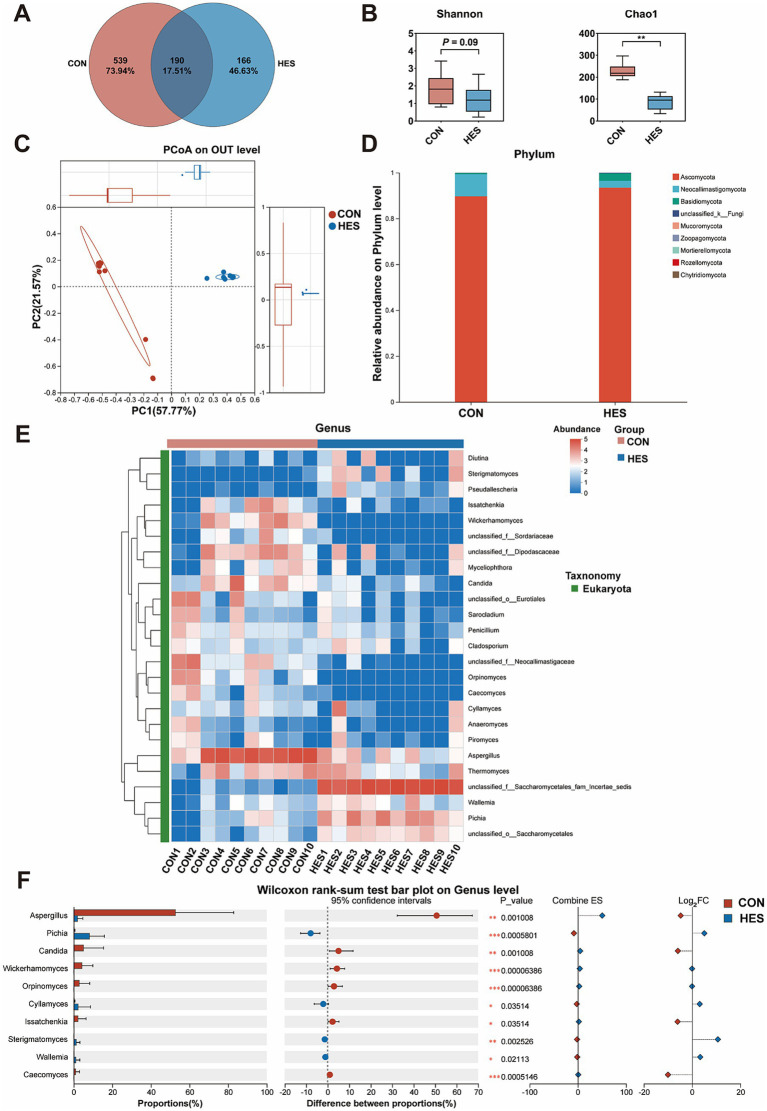
Effects of housed feeding pattern on the structure and composition of the fungal community in the rumen of Honghe cattle. **(A)** OUT Venn diagram. **(B)** α-diversity. **(C)** PCoA. **(D)** Map of fungal abundance at the phylum level. **(E)** Heatmap of Top 25 fungi at genus level. **(F)** Differences in genus level of fungi. Grazing group, i.e., control group (CON). Housed feeding group (HES).

We further examined the composition of the fungi. As shown in Figure 6, a total of 895 OUTs were identified, with the HES and CON groups occupying 539 OUTs and 166 OUTs, respectively (Figure 6A). The HES group significantly increased the Shannon and Chao1 indexes when compared with the CON group (Figure 6B) (*p* < 0.05). A significant difference between the HES and CON groups was found by PCoA analysis (Figure 6C). At the phylum level, Ascomycota, Neocallimastigomycota and Basidiomycota were the dominant phylum of rumen fungi and showed no significant difference between the HES and CON groups (Figure 6D). For the Top 25 fungi in terms of relative abundance at the genus level (Figure 6E), the HES group significantly enhanced the relative abundances of *Pichia*, *Cyllamyces*, *Sterigmatomyces* and *Wallemia* (*p* < 0.05), but decreased the relative abundances of *Aspergillus*, *Candida*, *Wickerhamomyces*, *Orpinomyces*, *Issatchenkia* and *Caecomyces* (Figure 6F) (*p* < 0.05).

### 3.6 Correlation analysis

**Figure 7:**
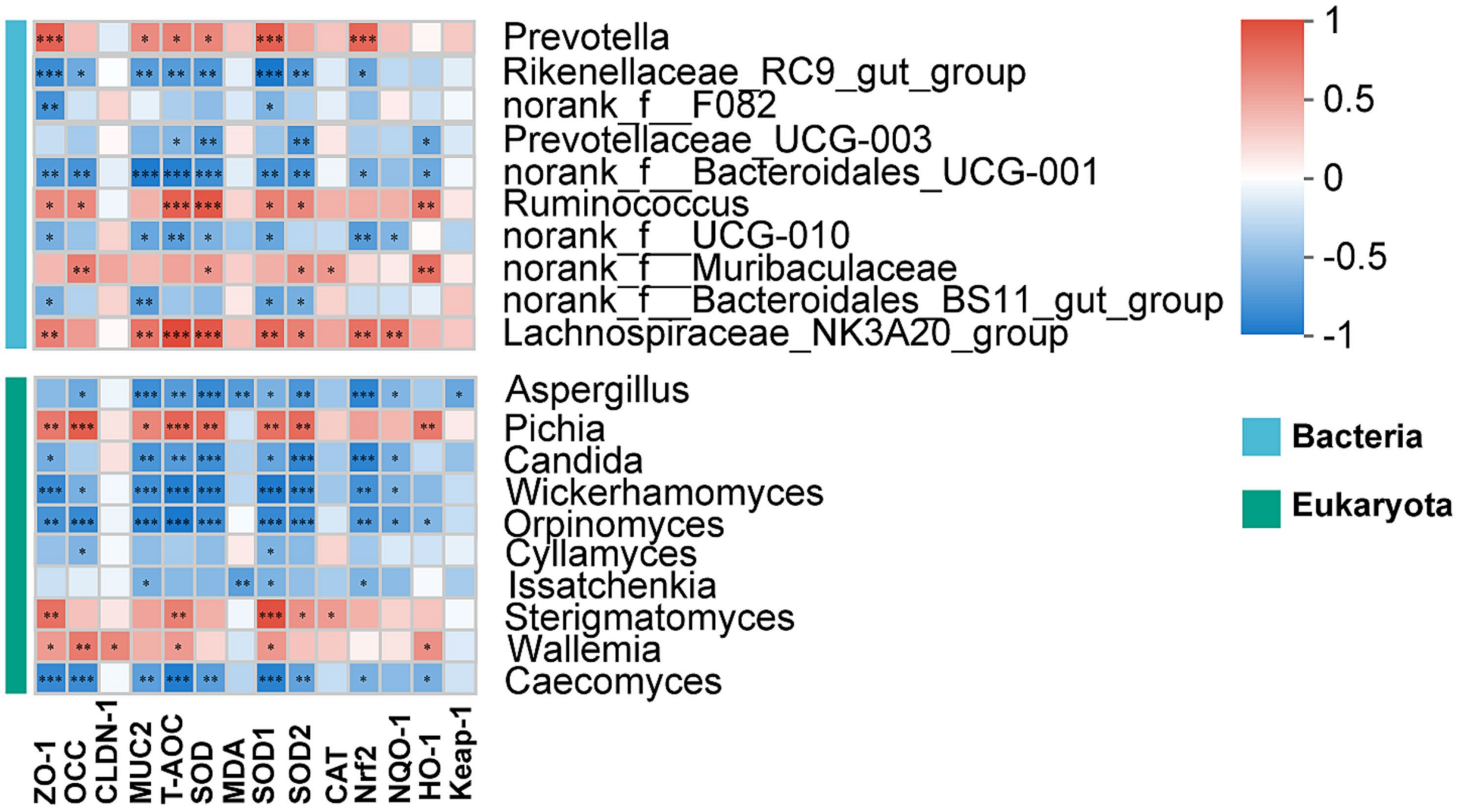
Correlation of rumen microbiota with rumen epithelial barrier and antioxidant capacity. *, **, and ***represent *p* < 0.05, *p* < 0.01, and *p* < 0.001, respectively.

As shown in Figure 7, we evaluated the correlation between rumen microbiota and the indicators related to rumen epithelial barrier and antioxidant capacity. The *ZO-1* was positively correlated with *Prevotella*, *Ruminococcus*, *Lachnospiraceae_NK3A20_group*, *Pichia*, *Sterigmatomyces* and *Wallemia* (*p* < 0.05). A positive correlation was observed between the levels of T-AOC and SOD and the *Prevotella*, *Ruminococcus*, *Lachnospiraceae_NK3A20_group* and *Pichia* (*p* < 0.05). The *SOD1* and *SOD2* both were positively correlated with *Ruminococcus*, *Lachnospiraceae_NK3A20_group*, *Pichia* and *Sterigmatomyces* (*p* < 0.05), but negatively correlated with *Rikenellaceae_RC9_gut_group*, *norank_f_Bacteroidales_UCG-001*, *norank_f_Bacteroidales_BS11_gut_group*, *Candida*, *Wickerhamomyces*, *Orpinomyces* and *Caecomyces* (*p* < 0.05). The CAT was positively correlated with *norank_f_Murbaculaceae* and *Sterigmatomyces* (*p* < 0.05). The Nrf2 was positively correlated *Prevotella* and *Lachnospiraceae_NK3A20_group* (*p* < 0.05).

## 4 Discussion

The sustainability of production performance in beef cattle may be challenged by climate, altitude, and management practices, although they can cope with a variety of survival conditions (7, 19). In particular, attention needs to be paid to changes in the physiological and metabolic profiles of beef cattle breeds that are undergoing a transition to intensive-housed feeding. In this study, housed feeding increased the concentrations of TP and ALB in the serum of Honghe cattle compared with grazing practice. There are many functions of circulating TP in the body, such as transporting various metabolites and regulating the physiological effects of the transported substances (20). The circulating TP content is significantly elevated to transport nutrients when protein metabolism is enhanced. Housed feeding provides a favorable environment for cattle to consume substances such as proteins, fats and carbohydrates. As a result, nutrient transport and metabolism are more vigorous in housed cattle than in grazing cattle. The ALB is one of the most important factors affecting many ligands in the blood circulation. Moreover, ALB synthesized by the liver has immunomodulatory functions, binds and transports many endogenous and exogenous substances, and enhances anti-inflammatory activity (21, 22). The hypoalbuminemia resulting from low ALB may be associated with many different diseases, including cirrhosis, nephrotic syndrome and malnutrition (23). Therefore, higher ALB within a healthy range implies better liver function, stable immunity and better nutrient delivery with housed feeding than grazing (24). Earlier study pointed out that an increase in serum glucose level represents a positive energy allocation after food intake in animals (15). In this study, elevated serum concentrations of GLU in housed cattle similarly confirmed positive nutritional metabolism. Thus, housed feeding contributes to the metabolic health of beef cattle.

Grazing environments may expose cattle to many different immune challenges including parasites, pathogenic bacteria and temperature stress when compared to housed environments (5). The environmental influences on the cattle immune system can be assessed by evaluating innate and adaptive immune indicators. Lejeune et al. (9) found that there were no significant differences between the peripheral and gastrointestinal immune systems of healthy cattle reared on outdoor pastures or indoors. In contrast, Di et al. (25) found that the short-term grazing of captive cows contributed to the activation of immune efficiency. These results are partly similar to those of Braghieri et al. (26), who reported a stronger immune system was observed in young bulls grazing for long periods. In our study, the serum concentrations of C3, C4, IL-4, IL-10, IL-1β and TNF-*α* were lower in the housed group than in the grazed group. Complement 3 and Complement 4 are important components of the complement system and are important participants in both innate and acquired immunity with the ability to enhance the ability of antibodies and phagocytes to remove microorganisms and damaged cells (27). Cytokines are small molecule proteins secreted by a variety of cells in the body and exert their biological effects by binding to the appropriate receptors on the cell surface (28). Normally, the inflammatory response of the body is inhibited by anti-inflammatory cytokines (e.g., IL-4 and IL-10), but activated by while pro-inflammatory factors (e.g., IL-1β and TNF-α) (11). However, both anti- and pro-inflammatory cytokines are increased during specific immune responses (29). For example, animal organisms need to maintain an active immune response to external environmental stresses. Therefore, in view of the results of our experiment, the cattle under grazing conditions were subjected to multiple stresses from the external environment, which resulted in the activation of the systemic immune response. Furthermore, animals possess complex antioxidant systems containing both enzymatic and non-enzymatic antioxidant systems, which act synergistically to protect animal cells and organ systems against free radical damage. The enzymatic antioxidant systems are represented by SOD and GSH-Px. Previous studies have found no significant effect of indoor feeding on the systematic antioxidant capacity of grazing ewes (8). At the present study, higher serum levels of T-AOC and SOD in housed compared to grazed cattle. It might be since the TMR substitution effect under housed conditions limited the uptake of antioxidant molecules from pasture grasses (30). The free radicals from pasture were not susceptible to uptake by housed cattle to induce oxidative stress. Notably, previous studies indicated a positive correlation between anti-inflammatory cytokines and antioxidant capacity (31). However, the concentration of IL-10 in serum decreased while the activity of SOD increased in the present experiment. This may be due to the fact that circulating concentrations of cytokines and antioxidant enzymes are affected by adaptive changes in tissues and organs and that this process is complex. Therefore, further analysis of immune and antioxidant responses in tissue organs (e.g., rumen) is necessary.

The rumen epithelium is constantly challenged by foreign antigens from pathogens and food and constitutes an important barrier to the external environment (10). At the tissue level, the rumen epithelium provided a defense system comprising commensal flora, immune, antioxidant, physical and chemical barriers that safeguarded nutrient uptake and protected the host from pathogenic organisms (11). To further assess whether systemic metabolism in cattle is related to rumen health, we first analyzed the immune response at the level of rumen tissue. No changes were observed in cytokine (IL-4, IL-10, IL-1β and TNF-*α*) concentrations in the rumen epithelium of housed feeding cattle compared to grazing cattle. Similarly, rumen immune function was unaffected by feeding practices at the level of gene expression. These results were inconsistent with the presence of a systemic immune response. For circulating immune factors, the effect of immune activation may be a synergistic effect of multiple organs such as rumen, liver and spleen (32–34). This implies that the effect of housed feeding on the immune response of cattle may not be related to the rumen. More research needs to be conducted to explain this phenomenon. It is worth noting that the antioxidant system, as an important component of rumen health, largely determines the ability of the rumen to defend itself against free radicals from pasture and feed (35). The SOD, GSH-Px, GSH and CAT are important antioxidant enzymes in the antioxidant system. In this experiment, we found that housed feeding cattle had higher levels of T-AOC and SOD in the rumen epithelium compared to grazing cattle. Additionally, we observed higher relative expression of *SOD1* and *SOD2* in the rumen epithelium of housed feeding cattle at the gene level. The expression of critical signaling factors such as *Nrf2*, *NQO-1* and *HO-1*, which determine the high expression of antioxidant enzymes, was also up-regulated under housed feeding condition. These results indicated that housed feeding cattle possess higher antioxidant capacity. It is possible that cattle are not exposed to excessive free radicals under stable nutritional intake conditions. There are irregularities in the grazing system, such as seasonal changes in the composition of the pasture zone (36). As a result, the nutrient intake of cattle cannot be maintained at a relatively constant level. In contrast, housed feeding cattle were allowed to feed freely and were not subject to environmental variability. From a nutritional point of view, it maintains a healthy metabolic state, resulting in a better rumen environment. Further assessing the effect of housing practices on rumen health in cattle at the RNA level, we found high expression of *ZO-1* and *OCC* genes in the rumen epithelium. With *ZO-1* and *OCC* generally contributing to the integrity of the gastrointestinal barrier, they are considered critical elements in the defense against exogenous infections, including pathogens and harmful antigens (11, 37). Housed feeding contributes to rumen development has been observed in the previous study of Huang et al. (6), as demonstrated by a more structurally intact rumen villus in housed yaks. This phenomenon was further illustrated in our trial, where the housed feeding facilitated the improvement of the rumen barrier function.

Undoubtedly, rumen microbial community is an important factor influencing ruminant growth and development. Previous studies indicated that the structure and composition of rumen microbiota were generally influenced by diet and feeding practices (6, 13, 38). Here, to systematically analyze the effects of different feeding practices on the rumen of Honghe cattle, we analyzed the bacterial and fungal communities of the rumen. The *α*-diversity indices (containing Shannon and Chao1) are comprehensive indicators for assessing the richness of microbial communities. A high level of α-diversity is closely related to the complexity and stability of bacterial composition and employed as a measure of bacterial resistance and adaptation to external perturbations. He et al. (13) indicated that the α-diversity of bacteria in the rumen of grazing yaks was higher than that of housed yak. Similarly, our study found that the α-diversity of rumen bacteria and fungi was higher in grazing conditions when compared with housed feeding cattle. This suggests that the abundance of rumen bacteria and fungi is higher in cattle under grazing conditions. Generally, the core bacterial and fungal communities are present in the rumen of beef cattle, although relative abundance may vary. In agreement with previous studies (39, 40), here, we found that Bacteroidata, Firmicutes and Proteobacteria constituted the core bacterial group of the rumen of Honghe cattle, whereas Ascomycota, Neocallimastigomycota and Basidiomycota constituted the core fungal group. According to previous studies, the rumen microbial community of ruminants was mainly composed of the Firmicutes and Bacteroidata, where Firmicutes was responsible for the decomposition of fibrous material, while Bacteroidata decomposed non-fibrous material (39, 41, 42). In contrast to previous study by He et al. (13), we found that housed feeding unaffected the above relative abundance of bacteria and fungi at the phylum level. This may be due to differences in cattle breeds and original grazing conditions. Notably, microbial communities are frequently affected by environmental conditions, including feeding practices and temperature. Moreover, previous studies focused mostly on the microbial response at the genus level in the rumen or feces. For instance, Zhang et al. reported that housed feeding increased the relative abundance of *Ruminococcaceae_UCG-010* and *Ruminococcaceae_UCG-013* in feces of cattle when compared with grazing group (43). Under relatively comfortable warm conditions, microorganisms such as *Prevotella*, *Bacteroides* and *Rikenellaceae_RC9_gut_group* in both rumen and feces exhibited high relative abundance (44). This shows that microorganisms at the genus level, both in the rumen and in the feces, undergo adaptive changes when faced with changes in environmental factors. Therefore, we further examined the changes in rumen microbiota at the genus level. Several microorganisms with high antioxidant activity will be present in the gastrointestinal tract, which can establish a set of antioxidant systems involved in the regulation of gastrointestinal health (45). *Prevotella* belongs to the family Prevotellacae and plays a crucial role in secreting enzymes and degrading starch as a core genus of Bacteroidetes (46). The substantial increase in the relative abundance of *Prevotella* was observed in study of fecal microbiota transplantation (FMT) for the treatment of inflammatory bowel diseases. This implies that *Prevotella* is one of the key microorganisms that benefit gastrointestinal health (47). Similar FMT intervention experiment also found that Ruminococcacae may play a role in modulating immune responses and inflammatory pathways in the gut. In our study, the relative abundances of *Prevotella* and *Ruminococcus* (a genus level within Ruminococcacae) were increased by housed feeding practices (48). Therefore, housed feeding may improve the health of the rumen of cattle by increasing beneficial bacteria such as *Prevotella* and *Ruminococcus*. Additionally, previous study showed a positive correlation between *Prevotella* and rumen antioxidant capacity (49). Similarly, the bacterium *norank_f_Muribaculaceae* also possessed a positive correlation with the antioxidant capacity of animal (50). Moreover, Yang et al. (38) found that dietary dioscorea opposite waste supplementation linearly increased the concentrations of T-AOC, SOD and GSH-Px in the serum of lambs and suggested that this may be related to an increase in *Ruminococcus*. Particularly, *Prevotella* and *Ruminococcus* were positively correlated with the levels of T-AOC and SOD and the expressions of *SOD1* and *SOD2*. For the gastrointestinal tract, previous studies have demonstrated that improved antioxidant capacity contributes to the integrity of barrier function. This implies that the improvement of rumen barrier function and antioxidant capacity in housed feeding cattle may be regulated by *Prevotella* and *Ruminococcus*. In this study, housed feeding also promoted the relative abundance *norank_f_Muribaculaceae* when compared with grazing. Furthermore, the effect of feeding practices on fungi in the rumen microbial system also requires attention. The diverse groups of dominant fungi may act synergistically in the rumen promoting rumen health. In agreement with Tong et al. (51), Geethanjali et al. (52) and Wang et al. (53), we identified Ascomycota, Neocallimastigomycota and Basidiomycota as the core fungal groups in the rumen and *Pichia* and *Cyllamyces* as the dominant genera in this study. *Pichia* is a dominant eukaryotic fungal genus in the rumen of ruminants (40). Previous study have found that Doenjang prepared with *Pichia* participation has antioxidant and neuroprotective effects and showed strong antioxidant activity in an *in vitro* cell culture model system (54). Several studies have also indicated that fungi such as *Pichia kudriavzevii* (55), *Pichia anomala* (56) and *Pichia pastoris* (57) possess antioxidant properties. The abundance of *Cyllamyces* was altered to accommodate the new diet composition when the rumen was exposed to nutritional stress (58). In the present study, housed feeding improved the relative abundance of *Pichia* and *Cyllamyces* when compared with traditional grazing patterns, suggesting that the elevated rumen antioxidant capacity may be related to the composition and abundance of the fungi. This speculation was supported by the positive correlation between Pichia and the concentrations of T-AOC and SOD in our experiment.

## 5 Conclusion

This study indicated that feeding mode was one of the critical factors influencing the healthiness of Honghe cattle. The housed feeding improved the metabolic status of the cattle and increased antioxidant capacity. Particularly, housed feeding improved rumen health by increasing barrier function and antioxidant capacity. The possible mechanism was that the housed feeding modulated the structure and composition of rumen bacteria and fungi. These results provided important insights for the large-scale breeding of beef cattle.

## Data Availability

The datasets presented in this study can be found in online repositories. The names of the repository/repositories and accession number(s) can be found at: https://www.ncbi.nlm.nih.gov/, PRJNA1206995, https://www.ncbi.nlm.nih.gov/, PRJNA1207129.

## References

[1] Nakajima N Doi K Tamiya S Yayota M. Effects of grazing in a sown pasture with forestland on the health of Japanese black cows as evaluated by multiple indicators. J Appl Anim Welf Sci. (2021) 24:173–87. doi: 10.1080/10888705.2020.1813581, PMID: 32877263

[2] Zhang J Gao Y Guo H Ding Y Ren W. Comparative metabolome analysis of serum changes in sheep under overgrazing or light grazing conditions. BMC Vet Res. (2019) 15:469. doi: 10.1186/s12917-019-2218-9, PMID: 31878922 PMC6933664

[3] Liu X Ding H Zhang X Ta N Zhao J Zhang Q. Dynamic changes in the gastrointestinal microbial communities of Gangba sheep and analysis of their functions in plant biomass degradation at high altitude. Microbiome. (2025) 13:17. doi: 10.1186/s40168-024-02022-5, PMID: 39838419 PMC11748513

[4] Bova T-L Chiavaccini L Cline G-F Hart C-G Matheny K Muth A-M. Environmental stressors influencing hormones and systems physiology in cattle. Reprod Biol Endocrinol. (2014) 12:58. doi: 10.1186/1477-7827-12-58, PMID: 24996419 PMC4094414

[5] Cooke A-S Mullan S Morten C Hockenhull J Le Grice P Le Cocq K. Comparison of the welfare of beef cattle in housed and grazing systems: hormones, health, and behaviour. J Agric Sci. (2023) 161:450–63. doi: 10.1017/S0021859623000357, PMID: 37641790 PMC7614983

[6] Huang C Ge F Yao X Guo X Bao P Ma X. Microbiome and metabolomics reveal the effects of different feeding systems on the growth and ruminal development of yaks. Front Microbiol. (2021) 12:682989. doi: 10.3389/fmicb.2021.682989, PMID: 34248900 PMC8265505

[7] Abebe B-K Wang J Guo J Wang H Li A Zan L. A review of emerging technologies, nutritional practices, and management strategies to improve intramuscular fat composition in beef cattle. Anim Biotechnol. (2024) 35:2388704. doi: 10.1080/10495398.2024.2388704, PMID: 39133095 PMC12674276

[8] Jin Y-M Zhang X-Q Badgery W-B Li P Wu JX. Effects of winter and spring housing on growth performance and blood metabolites of Pengbo semi-wool sheep in Tibet. Asian Australas J Anim Sci. (2019) 32:1630–9. doi: 10.5713/ajas.18.0966, PMID: 31010990 PMC6718902

[9] Lejeune A Monahan F-J Moloney A-P Earley B Black A-D Campion D-P. Peripheral and gastrointestinal immune systems of healthy cattle raised outdoors at pasture or indoors on a concentrate-based ration. BMC Vet Res. (2010) 6:19. doi: 10.1186/1746-6148-6-19, PMID: 20356390 PMC2864234

[10] Baldwin R-L Connor E-E. Rumen function and development. Vet Clin North Am Food Anim Pract. (2017) 33:427–39. doi: 10.1016/j.cvfa.2017.06.001, PMID: 28807474

[11] Zou B Long F Xue F Chen C Zhang X Qu M. Protective effects of niacin on rumen epithelial cell barrier integrity in heat-stressed beef cattle. Animals. (2024) 14:14. doi: 10.3390/ani14020313, PMID: 38275773 PMC10812637

[12] Zhang J Shi H Wang Y Li S Cao Z Ji S. Effect of dietary forage to concentrate ratios on dynamic profile changes and interactions of ruminal microbiota and metabolites in Holstein heifers. Front Microbiol. (2017) 8:2206. doi: 10.3389/fmicb.2017.02206, PMID: 29170660 PMC5684179

[13] He S Yuan Z Dai S Wang Z Zhao S Wang R. Intensive feeding alters the rumen microbiota and its fermentation parameters in natural grazing yaks. Front Vet Sci. (2024) 11:1365300. doi: 10.3389/fvets.2024.1365300, PMID: 38645650 PMC11027562

[14] Han L Yu Y Fu R Fu B Gao H Li Z. Impact of various ration energy levels on the slaughtering performance, carcass characteristics, and meat qualities of Honghe yellow cattle. Food Secur. (2024) 13:13. doi: 10.3390/foods13091316, PMID: 38731687 PMC11083055

[15] Fu R Liang C Chen D Tian G Zheng P He J. Effects of low-energy diet supplemented with betaine on growth performance, nutrient digestibility and serum metabolomic profiles in growing pigs. J Anim Sci. (2023) 101:skad080. doi: 10.1093/jas/skad080, PMID: 36930062 PMC10066726

[16] Arce R Barros S Wacker B Peters B Moss K Offenbacher S. Increased TLR4 expression in murine placentas after oral infection with periodontal pathogens. Placenta. (2009) 30:156–62. doi: 10.1016/j.placenta.2008.11.017, PMID: 19101032 PMC2656361

[17] Liu H Ran T Zhang C Yang W Wu X Degen A. Comparison of rumen bacterial communities between yaks (*Bos grunniens*) and Qaidam cattle (*Bos taurus*) fed a low protein diet with different energy levels. Front Microbiol. (2022) 13:982338. doi: 10.3389/fmicb.2022.982338, PMID: 36147854 PMC9486477

[18] Wu J Tian C Jiao J Yan Q Zhou C Tan Z. The epithelial transcriptome and mucosal microbiota are altered for goats fed with a low-protein diet. Front Microbiol. (2023) 14:1237955. doi: 10.3389/fmicb.2023.1237955, PMID: 37731924 PMC10507412

[19] Lyu Y Wang F Cheng H Han J Dang R Xia X. Recent selection and introgression facilitated high-altitude adaptation in cattle. Sci Bull. (2024) 69:3415–24. doi: 10.1016/j.scib.2024.05.030, PMID: 38945748

[20] Aghakhani M Shahraki A-D-F Tabatabaei S-N Toghyani M Moosavi-Zadeh E Rafiee H. 24-hour postnatal total serum protein concentration affects the health and growth performance of female Holstein dairy calves. Vet Med Sci. (2023) 9:2230–7. doi: 10.1002/vms3.1203, PMID: 37459751 PMC10508483

[21] Rozga J Piątek T Małkowski P. Human albumin: old, new, and emerging applications. Ann Transplant. (2013) 18:205–17. doi: 10.12659/AOT.889188, PMID: 23792522

[22] Caraceni P Domenicali M Tovoli A Napoli L Ricci CS Tufoni M. Clinical indications for the albumin use: still a controversial issue. Eur J Intern Med. (2013) 24:721–8. doi: 10.1016/j.ejim.2013.05.015, PMID: 23790570

[23] Soeters P-B Wolfe R-R Shenkin A. Hypoalbuminemia: pathogenesis and clinical significance. JPEN J Parenter Enteral Nutr. (2019) 43:181–93. doi: 10.1002/jpen.1451, PMID: 30288759 PMC7379941

[24] Garcia-Martinez R Caraceni P Bernardi M Gines P Arroyo V Jalan R. Albumin: pathophysiologic basis of its role in the treatment of cirrhosis and its complications. Hepatology. (2013) 58:1836–46. doi: 10.1002/hep.26338, PMID: 23423799

[25] Di Grigoli A Di Trana A Alabiso M Maniaci G Giorgio D Bonanno A. Effects of grazing on the behaviour, oxidative and immune status, and production of organic dairy cows. Animals. (2019) 9:9. doi: 10.3390/ani9060371, PMID: 31216727 PMC6617352

[26] Braghieri A Pacelli C De Rosa G Girolami A De Palo P Napolitano F. Podolian beef production on pasture and in confinement. Animal. (2011) 5:927–37. doi: 10.1017/S1751731110002685, PMID: 22440032

[27] Vorup-Jensen T Jensen R-K. Structural immunology of complement receptors 3 and 4. Front Immunol. (2018) 9:2716. doi: 10.3389/fimmu.2018.02716, PMID: 30534123 PMC6275225

[28] Kany S Vollrath J-T Relja B. Cytokines in inflammatory disease. Int J Mol Sci. (2019) 20:6008. doi: 10.3390/ijms20236008, PMID: 31795299 PMC6929211

[29] Fu R Liang C Chen D Yan H Tian G Zheng P. Effects of dietary *Bacillus coagulans* and yeast hydrolysate supplementation on growth performance, immune response and intestinal barrier function in weaned piglets. J Anim Physiol An N. (2021) 105:898–907. doi: 10.1111/jpn.13529, PMID: 33715204

[30] Lee S-H Jew S-S Chang P-S Hong I-J Hwang E-S Kim K-S. Free radical scavenging effect and antioxidant activities of barley leaves. Food Sci Biotechnol. (2003) 12:268–73.

[31] Chen J Guo Q Chen Q Chen Y Chen D Chen Z. Interleukin 10 inhibits oxidative stress-induced autophagosome formation in hepatic stellate cells by activating the mTOR-STAT3 pathway. Exp Cell Res. (2022) 411:113001. doi: 10.1016/j.yexcr.2021.113001, PMID: 34973945

[32] Kubes P Jenne C. Immune responses in the liver. Annu Rev Immunol. (2018) 36:247–77. doi: 10.1146/annurev-immunol-051116-052415, PMID: 29328785

[33] Lewis S-M Williams A Eisenbarth S-C. Structure and function of the immune system in the spleen. Sci Immunol. (2019) 4:4. doi: 10.1126/sciimmunol.aau6085, PMID: 30824527 PMC6495537

[34] Gozho G-N Krause D-O Plaizier J-C. Ruminal lipopolysaccharide concentration and inflammatory response during grain-induced subacute ruminal acidosis in dairy cows. J Dairy Sci. (2007) 90:856–66. doi: 10.3168/jds.S0022-0302(07)71569-2, PMID: 17235162

[35] Izuddin W-I Humam A-M Loh T-C Foo H-L Samsudin A-A. Dietary postbiotic *Lactobacillus plantarum* improves serum and ruminal antioxidant activity and upregulates hepatic antioxidant enzymes and ruminal barrier function in post-weaning lambs. Antioxidants. (2020) 9:250. doi: 10.3390/antiox9030250, PMID: 32204511 PMC7139658

[36] Șanta A Mierlita D Dărăban S Socol C-T Vicas S-I Șuteu M. The effect of sustainable feeding systems, combining total mixed rations and pasture, on milk fatty acid composition and antioxidant capacity in Jersey dairy cows. Animals. (2022) 12:12. doi: 10.3390/ani12070908, PMID: 35405896 PMC8997149

[37] Kuo W-T Zuo L Odenwald M-A Madha S Singh G Gurniak CB. The tight junction protein ZO-1 is dispensable for barrier function but critical for effective mucosal repair. Gastroenterology. (2021) 161:1924–39. doi: 10.1053/j.gastro.2021.08.047, PMID: 34478742 PMC8605999

[38] Yang R Guo Y Zhang S Hao Q Duan C Wang Y. Effect of dioscorea opposite waste supplementation on antioxidant capacity, immune response and rumen microbiome in weaned lambs. Fermentation. (2023) 9:256. doi: 10.3390/fermentation9030256

[39] Bi Y Zeng S Zhang R Diao Q Tu Y. Effects of dietary energy levels on rumen bacterial community composition in Holstein heifers under the same forage to concentrate ratio condition. BMC Microbiol. (2018) 18:69. doi: 10.1186/s12866-018-1213-9, PMID: 29996759 PMC6042446

[40] Li L Qu L Li T. The effects of Selenohomolanthionine supplementation on the rumen eukaryotic diversity of Shaanbei white cashmere wether goats. Sci Rep. (2023) 13:13134. doi: 10.1038/s41598-023-39953-2, PMID: 37573461 PMC10423290

[41] Evans N-J Brown J-M Murray R-D Getty B Birtles RJ Hart CA. Characterization of novel bovine gastrointestinal tract Treponema isolates and comparison with bovine digital dermatitis treponemes. Appl Environ Microbiol. (2011) 77:138–47. doi: 10.1128/AEM.00993-10, PMID: 21057019 PMC3019712

[42] Reigstad C-S Kashyap P-C. Beyond phylotyping: understanding the impact of gut microbiota on host biology. Neurogastroenterol Motil. (2013) 25:358–72. doi: 10.1111/nmo.12134, PMID: 23594242 PMC4524550

[43] Zhang Z Yang L He Y Luo X Zhao S Jia X. Composition of fecal microbiota in grazing and feedlot Angus beef cattle. Animals. (2021) 11:11. doi: 10.3390/ani11113167, PMID: 34827898 PMC8614352

[44] Luo T Zhu J Li K Li Y Li J Chen Y. Crosstalk between innate immunity and rumen-fecal microbiota under the cold stress in goats. Front Immunol. (2024) 15:1363664. doi: 10.3389/fimmu.2024.1363664, PMID: 38476231 PMC10928366

[45] Wu L Xie X Li Y Liang T Zhong H Yang L. Gut microbiota as an antioxidant system in centenarians associated with high antioxidant activities of gut-resident *Lactobacillus*. Npj Biofilms Microbi. (2022) 8:102. doi: 10.1038/s41522-022-00366-0, PMID: 36564415 PMC9789086

[46] Flint H-J Bayer E-A Rincon M-T Lamed R White B-A. Polysaccharide utilization by gut bacteria: potential for new insights from genomic analysis. Nat Rev Microbiol. (2008) 6:121–31. doi: 10.1038/nrmicro1817, PMID: 18180751

[47] He R Li P Wang J Cui B Zhang F Zhao F. The interplay of gut microbiota between donors and recipients determines the efficacy of fecal microbiota transplantation. Gut Microbes. (2022) 14:2100197. doi: 10.1080/19490976.2022.2100197, PMID: 35854629 PMC9302524

[48] Rees NP Shaheen W Quince C Tselepis C Horniblow RD Sharma N. Systematic review of donor and recipient predictive biomarkers of response to faecal microbiota transplantation in patients with ulcerative colitis. EBioMedicine. (2022) 81:104088. doi: 10.1016/j.ebiom.2022.104088, PMID: 35660786 PMC9163485

[49] Wu Q Chen H Zhang F Wang W Xiong F Liu Y. Cysteamine supplementation in vitro remarkably promoted rumen fermentation efficiency towards propionate production via *Prevotella* enrichment and enhancing antioxidant capacity. Antioxidants. (2022) 11:11. doi: 10.3390/antiox11112233, PMID: 36421419 PMC9686782

[50] Fang W Peng W Qi W Zhang J Song G Pang S. Ferulic acid combined with different dietary fibers improve glucose metabolism and intestinal barrier function by regulating gut microbiota in high-fat diet-fed mice. J Funct Foods. (2024) 112:105919. doi: 10.1016/j.jff.2023.105919

[51] Tong Y Wu J Guo W Yang Z Wang H Liu H. The effect of combining millet and corn straw as source forage for beef cattle diets on ruminal degradability and fungal community. Animals (Basel). (2023) 13:548. doi: 10.3390/ani13040548, PMID: 36830335 PMC9951761

[52] Geethanjali K Rathnagiri P Kalarani V. Heterologous expression of cysteine protease 8 from Trichomonas foetus in Pichia pastoris. J Vet Ani Sci. (2023) 54:13–20. doi: 10.51966/jvas.2023.54.1.13-20

[53] Wang H Li P Liu X Zhang C Lu Q Xi D. The composition of fungal communities in the rumen of gayals (*Bos frontalis*), yaks (*Bos grunniens*), and Yunnan and Tibetan yellow cattle (*Bos taurs*). Pol J Microbiol. (2019) 68:505–14. doi: 10.33073/pjm-2019-050, PMID: 31880894 PMC7260705

[54] Kang S-J Seo J-Y Cho K-M Lee C-K Kim J-H Kim JS. Antioxidant and neuroprotective effects of doenjang prepared with *Rhizopus*, *Pichia*, and *Bacillus*. Prev Nutr Food Sci. (2016) 21:221–6. doi: 10.3746/pnf.2016.21.3.221, PMID: 27752498 PMC5063207

[55] Chi M Li G Liu Y Liu G Li M Zhang X. Increase in antioxidant enzyme activity, stress tolerance and biocontrol efficacy of *Pichia kudriavzevii* with the transition from a yeast-like to biofilm morphology. Biol Control. (2015) 90:113–9. doi: 10.1016/j.biocontrol.2015.06.006

[56] Chen Y Wan Y Cai W Liu N Zeng J Liu C. Effects on cell membrane integrity of *Pichia anomala* by the accumulating excessive reactive oxygen species under ethanol stress. Food Secur. (2022) 11:11. doi: 10.3390/foods11223744, PMID: 36429336 PMC9689904

[57] Yazdi FT Tanhaeian A Azghandi M Vasiee A Alizadeh Behbahani B Mortazavi SA. Heterologous expression of Thrombocidin-1 in *Pichia pastoris*: evaluation of its antibacterial and antioxidant activity. Microb Pathog. (2019) 127:91–6. doi: 10.1016/j.micpath.2018.11.047, PMID: 30513368

[58] Yang X Fan X Jiang H Zhang Q Zhang Q Dang S. Simulated seasonal diets alter yak rumen microbiota structure and metabolic function. Front Microbiol. (2022) 13:1006285. doi: 10.3389/fmicb.2022.1006285, PMID: 36212853 PMC9538157

