# Supplementary material for: Housed feeding improves rumen health by influencing the composition of the microbiota in Honghe cattle
Source: Front Vet Sci. 2025 Mar 10;12:1556934. doi: 10.3389/fvets.2025.1556934 (PMC11931650; doi:10.3389/fvets.2025.1556934)
Supplement: Supplementary file 1 [file Table_1.docx]

Supplementary Material

Supplementary TABLE S1 The basic components of the TMR in housed feeding group

| Ingredients | Content (%) |
| --- | --- |
| Corn | 47.5 |
| Wheat Bran | 5.73 |
| Rapeseed Cake | 6.32 |
| Baking Soda | 0.20 |
| Premix Compound | 0.20 |
| Corn silage | 25.00 |
| Oat Hay | 17.00 |
| Total | 100.00 |

Supplementary TABLE S2 Primer sequences used for real-time PCR

| Items | Primer sequence (5’ –3’) | Product length(bp) | Accession number |
| --- | --- | --- | --- |
| *ACTB* | F: GCGGCATTCACGAAACTACC | 148 | [NM_173979.3](https://www.ncbi.nlm.nih.gov/entrez/viewer.fcgi?db=nucleotide&id=75832053) |
|  | R: GCCAGGGCAGTGATCTCTTT |  |  |
| *OCC* | F: TCGACCAATGCTCTCTCAGC | 196 | [XM_024981258.2](https://www.ncbi.nlm.nih.gov/entrez/viewer.fcgi?db=nucleotide&id=2587656720) |
|  | R: ACCAGCTCCCATTAAGGTTCC |  |  |
| *ZO-1* | F: TGGAATGCATCCTGACCACC | 113 | XM_024982009.2 |
|  | R: TGTACACCTGTTGAGAGGCT |  |  |
| *CLDN-1* | F: GGCAGATCCAGTGCAAAGTC | 83 | [XM_061413067.1](https://www.ncbi.nlm.nih.gov/entrez/viewer.fcgi?db=nucleotide&id=2633682857) |
|  | R: GCCAATCACCATCAAGGCAC |  |  |
| *MUC2* | F: TTGTGTCCTACAACGGCCTC | 141 | [XM_024987595.2](https://www.ncbi.nlm.nih.gov/entrez/viewer.fcgi?db=nucleotide&id=2587674241) |
|  | R: AGTTGTCAATGCTCTCCCCG |  |  |
| *IL-4* | F: GCTGCCCCAAAGAACACAAC | 77 | [NM_173921.2](https://www.ncbi.nlm.nih.gov/entrez/viewer.fcgi?db=nucleotide&id=31343261) |
|  | R: TGGCTCCTGTAGATACGCCT |  |  |
| *IL-10* | F: TGATGCCACAGGCTGAGAAC | 126 | [NM_174088.1](https://www.ncbi.nlm.nih.gov/entrez/viewer.fcgi?db=nucleotide&id=41386771) |
|  | R: TCTTGTTTTCGCAGGGCAGA |  |  |
| *IL-1β* | F: AAAAGCTTCAGGCAGGTGGT | 155 | [NM_174093.1](https://www.ncbi.nlm.nih.gov/entrez/viewer.fcgi?db=nucleotide&id=27806570) |
|  | R: AACTCGTCGGAGGACGTTTC |  |  |
| *TNF-α* | F: AAGTAACAAGCCGGTAGCCC | 150 | [XM_005223596.5](https://www.ncbi.nlm.nih.gov/entrez/viewer.fcgi?db=nucleotide&id=2587662267) |
|  | R: ATGAGGTAAAGCCCGTCAGC |  |  |
| *TRAF6* | F: TGCGCCAACTACATATCGCT | 121 | [XM_005216384.5](https://www.ncbi.nlm.nih.gov/entrez/viewer.fcgi?db=nucleotide&id=2587636743) |
|  | R: TCACAGCTGCTTCAGACTGG |  |  |
| *TLR4* | F: AAAAAGTATGGCAGGGGCGA | 77 | [NM_174198.6](https://www.ncbi.nlm.nih.gov/entrez/viewer.fcgi?db=nucleotide&id=126723186) |
|  | R: TTCCGCACCCAGTCTTCATC |  |  |
| *MyD88* | F: ATTGAGAAGAGGTGCCGTCG | 189 | [NM_001014382.2](https://www.ncbi.nlm.nih.gov/entrez/viewer.fcgi?db=nucleotide&id=75832100) |
|  | R: ACAGACAGTGATGAAGCGCA |  |  |
| *NF-kB* | F: GCAGCTGCAGTTTGATACCG | 95 | [NM_001080242.2](https://www.ncbi.nlm.nih.gov/entrez/viewer.fcgi?db=nucleotide&id=126723253) |
|  | R: AGTTGTCGACAGATGCCAGG |  |  |
| *SOD1* | F: AAAACGGTGTTGCCATCGTG | 121 | [NM_174615.2](https://www.ncbi.nlm.nih.gov/entrez/viewer.fcgi?db=nucleotide&id=31341527) |
|  | R: TTTCCACCTCTGCCCAAGTC |  |  |
| *SOD2* | F: CGCTGGAGAAGGGTGATGTT | 156 | [NM_201527.2](https://www.ncbi.nlm.nih.gov/entrez/viewer.fcgi?db=nucleotide&id=88853815) |
|  | R: CACGTTTGATGGCTTCCAGC |  |  |
| *GSH-Px* | F: AACGTAGCATCGCTCTGAGG | 121 | [XM_019984632.1](https://www.ncbi.nlm.nih.gov/entrez/viewer.fcgi?db=nucleotide&id=1131226315) |
|  | R: GATGCCCAAACTGGTTGCAG |  |  |
| *CAT* | F: GAGGAAACGCCTGTGTGAGA | 116 | [NM_001035386.2](https://www.ncbi.nlm.nih.gov/entrez/viewer.fcgi?db=nucleotide&id=402693375) |
|  | R: GGATGCGGGAGCCATATTCA |  |  |
| *Nrf2* | F: GTCCCAGCAGGACATGGATT | 107 | [NM_001011678.2](https://www.ncbi.nlm.nih.gov/entrez/viewer.fcgi?db=nucleotide&id=147904941) |
|  | R: GCTCATGCTCCTTCTGTCGT |  |  |
| *NQO-1* | F: ACCAACAGACCAGCCAATCA | 113 | [XM_061387766.1](https://www.ncbi.nlm.nih.gov/entrez/viewer.fcgi?db=nucleotide&id=2633757282) |
|  | R: GCAGCCTCCTTCATGGCATA |  |  |
| *HO-1* | F: CAAGCGCTATGTTCAGCGAC | 198 | [NM_001014912.1](https://www.ncbi.nlm.nih.gov/entrez/viewer.fcgi?db=nucleotide&id=62460519) |
|  | R: TTGGTGGCACTGGCGATATT |  |  |
| *Keap-1* | F: AGAGAAACGAGTGGCGGATG | 159 | [XM_005208764.4](https://www.ncbi.nlm.nih.gov/entrez/viewer.fcgi?db=nucleotide&id=2587690311) |
|  | R: ACGTCCACGTTTCTGTCTCC |  |  |

ACTB (β-actin), zonula occludens-1 (ZO1), occludin (OCC), claudin-1 (CLDN-1), mucin2 (MUC2), interleukin-4 (IL-4), interleukin-10 (IL-10), interleukin-1β (IL-1β), tumor necrosis factor α (TNF-α), TNF receptor associated factor 6 (TRAF6), toll-like receptor 4 (TLR4), myeloid differentiation factor-88 (MyD88) and nuclear factor kappa-B (NF-κB), superoxide dismutase 1 (SOD1), superoxide dismutase 2 (SOD2), glutathione peroxidase (GSH-Px), catalase (CAT), nuclear factor erythroid 2-related factor 2 (Nrf2), NAD(P)H:quinone oxidoreductase 1 (NQO-1), heme oxygenase-1 (HO-1) and kelch-like-ECH associated protein-1 (Keap-1).
